# Immunity to SARS-CoV-2: Lessons Learned

**DOI:** 10.3389/fimmu.2021.654165

**Published:** 2021-03-19

**Authors:** Jaime Fergie, Amit Srivastava

**Affiliations:** ^1^Department of Pediatric Infectious Diseases, Driscoll Children's Hospital, Corpus Christi, TX, United States; ^2^Vaccine Medical Development, Scientific and Clinical Affairs, Pfizer Inc, Collegeville, PA, United States

**Keywords:** immunity, COVID-19, SARS-CoV-2, duration of protection, receptor-binding domain, spike protein, vaccination, systems serology

## Abstract

In the year since the emergence of severe acute respiratory syndrome coronavirus 2 (SARS-CoV-2) and with understanding of the etiology of the coronavirus disease 2019 (COVID-19) pandemic, it has become clear that most infected individuals achieve some form of immunity against the virus with relatively few reported reinfections. A number of vaccines have already achieved emergency use authorization based on data from large phase 3 field efficacy clinical trials. However, our knowledge about the extent and durability of this immunity, and the breadth of vaccine coverage against SARS-CoV-2 variants is still evolving. In this narrative review, we summarize the latest and rapidly developing understanding of immunity to SARS-CoV-2 infection, including what we have learned about the key antigens of SARS-CoV-2 (i.e., the spike protein and its receptor-binding domain), their importance in vaccine development, the immediate immune response to SARS-CoV-2, breadth of coverage of emerging SARS-CoV-2 variants, contributions of preexisting immunity to related coronaviruses, and duration of immunity. We also discuss lessons from newer approaches, such as systems serology, that provide insights into molecular and cellular immune responses elicited and how they relate to the trajectory of infection, and potentially inform immune correlates of protection. We also briefly examine the limited research literature on immune responses in special populations, such as pregnant women and children.

## Introduction

In December 2019 in Wuhan, China, a novel coronavirus (CoV) emerged, causing severe acute respiratory syndrome (SARS) in humans (1). The virus, SARS coronavirus 2 (SARS-CoV-2), causes coronavirus disease 2019 (COVID-19), which has subsequently caused a global pandemic (2). As of February 15, 2021, >108 million cases of COVID-19 and almost 2.4 million deaths have been reported worldwide (3).

Seven CoVs are known to infect humans: 4 seasonal CoVs that cause self-limiting upper respiratory tract infections and 3 highly pathogenic CoVs (i.e., SARS coronavirus [SARS-CoV-1], Middle East respiratory syndrome [MERS], and SARS-CoV-2), which emerged in 2003, 2012, and 2019, respectively (4, 5). Knowledge of immunity to all CoVs is sparse (4). Immunity to the seasonal CoVs appears to last ~1 year, whereas for patients with SARS-CoV-1 and MERS, antibody levels are greatly reduced 2–3 years after symptom onset, indicating that patients might be susceptible to reinfection at that time (6–8). Less than a year into the COVID-19 pandemic, our understanding of immunity to SARS-CoV-2 is developing rapidly. A substantial portion of our current knowledge is derived from serosurveillance studies conducted in the very early stages of the pandemic and subsequently from evaluations of B- and T-cell responses among convalescent patients with varying degrees of disease severity. Hospitalized patients who eventually died of COVID-19 have also been analyzed to help correlate the immune response with disease trajectory. In addition, several studies have evaluated sera from the prepandemic period and from longitudinal studies with follow-up from studies on the SARS-CoV-1 pandemic and other CoVs. Inevitably, there are several unanswered questions; however, a remarkable body of knowledge has rapidly accumulated from the scientific enterprise during this pandemic, yielding actionable insights that continue to inform development of vaccines and therapeutics to combat COVID-19. As of this writing, there are ~60 vaccine candidates at various stages of clinical trials and about 172 candidates in preclinical development spanning diverse vaccine platforms, such as inactivated and live virus, protein subunits, viral vector, DNA, and mRNA (9). In a remarkable, unprecedented scientific achievement, after only <10 months in clinical development, 2 vaccine candidates have reported ~95% vaccine efficacy (VE) against COVID-19 from large (>30,000 subjects) prospective placebo-controlled phase 3 clinical trials (10, 11). Before the end of 2020, both vaccines were granted emergency use authorization (EUA) by the US Food and Drug Administration (FDA) (12, 13), and conditional marketing authorization by the European Medicines Agency (14, 15), with vaccinations currently underway in multiple countries. Adenovirus (Ad)-based vaccines in late-stage clinical trials have reported efficacies of 62−92%, depending on the vaccine, dosing regimen, and disease severity (16–18); the ChAdOx-1 nCov-19 vaccine has been authorized for use during the pandemic in the United Kingdom, and in several European and other countries, and vaccinations are in progress (19–22). Interim analysis of a late-stage protein-based vaccine trial reported 89% efficacy against COVID-19 (with lower VE against disease caused by emerging SARS-CoV-2 variants) (23). Finally, multiple whole inactivated SARS-CoV-2 vaccines currently in clinical trials have reported promising safety and immunogenicity findings (24–27). In addition, numerous SARS-CoV-2 neutralizing monoclonal antibodies (mAbs) have been derived from convalescent patient sera and are being evaluated in clinical trials, and 2 mAb therapeutics have been also granted EUA by the FDA (28, 29).

Although it is becoming clear that most infected individuals achieve some form of immunity against COVID-19, with extremely few reported reinfections (30–32), the extent and durability of this immunity, including breadth of coverage of SARS-CoV-2 variants, are among the key research questions. Addressing these queries will guide research into potential vaccines and immunotherapeutics and inform vaccination recommendations from national vaccine technical committees. Therefore, the aim of this narrative review is to summarize the latest understanding of immunity to SARS-CoV-2 infection. From a rapidly advancing body of scientific literature, we examine the key antigens of SARS-CoV-2 and what is known about the immediate immune response to SARS-CoV-2 infection, the contributions of preexisting immunity to related CoVs, the breadth of immune coverage of emerging SARS-CoV-2 variants, and the duration of immunity. We also discuss newer approaches such as systems serology that can provide insights into the molecular and cellular immune responses in relation to the trajectory of infection and potentially inform immune correlates of protection. Finally, we examine the limited research literature on immune responses in special populations, such as pregnant women and children.

## Methods

A targeted literature search was performed using PubMed databases. We used the search term “coronavirus” and each of the following terms or phrases singly and in combination: “SARS-CoV-2,” “immunity,” “systems serology,” “cellular immunity,” “duration of protection,” “breadth of coverage,” and “vaccination.” We also searched for each of these terms with the search terms “SARS” and “MERS.” References cited within the retrieved publications were used as appropriate. The search was limited to English-language articles published up to December 31, 2020. We included ahead-of-print publications available in the PubMed database. Additionally, references from the authors' personal files were reviewed.

## Key Antigens of SARS-CoV-2

It is now understood that the dominant antigen is the SARS-CoV-2 spike protein, which *via* its receptor-binding domain (RBD) is responsible for binding the virus to the human angiotensin-converting enzyme 2 (ACE2) receptor and its subsequent cellular uptake (33–35).

One of the earliest studies to characterize the humoral responses to SARS-CoV-2 infection used HIV-1–based virions pseudotyped with SARS-CoV-2 spike protein (SARS-CoV-2 pseudovirus) to evaluate the human antibody response to SARS-CoV-2 in 149 individuals recovering from COVID-19 of varying severity (36). Neutralization of SARS-CoV-2 pseudovirus by plasma from convalescent patients with COVID-19, collected on an average of 39 days after the onset of symptoms, had variable half-maximal neutralizing titers (NT_50_). In 33% of the samples, NT_50_ values were <50, and in 79% of the samples, they were <1,000. It is interesting to contemplate the parallels here with data from non-human primate challenge studies, in which low titers (1:20 and above) of circulating neutralizing antibodies were reported in animals protected from COVID-19 (37). Antibody cloning showed expanded clones of RBD-specific memory B cells expressing closely related antibodies in different individuals. Despite low plasma NT_50_ values (e.g., 297–10,433), antibodies to 3 distinct epitopes on the RBD neutralized the pseudovirus at half-maximal inhibitory concentrations (IC_50_) ranging from 1.6 to 3.0 ng/mL (36). This study was the earliest report noting no significant cross-reactivity to the RBDs of other key CoVs, such as MERS or human coronaviruses HCoV-OC43,−229E, or NL63 (36). Similar findings regarding neutralizing titers were reported in a cross-sectional study of hospitalized patients acutely infected with COVID-19 (*n* = 44): RBD-specific immunoglobulin G (IgG) responses and neutralizing titers were detectable in all patients within 6 days after confirmation with polymerase chain reaction (PCR) (38). Additionally, the magnitude of RBD-specific IgG titers was positively correlated with the neutralizing potency (38).

A study that screened and selected convalescent patient sera with high neutralizing titers against SARS-CoV-2 found that most epitopes targeted the spike RBD (39). Pseudovirus-neutralizing titers varied for SARS-CoV-2 and were low to undetectable against SARS-CoV-1, whereas anti-RBD mAbs were of extremely high potency, down to 0.019 μg/mL (39). Similar findings were reported by Piccoli and colleagues in which SARS-CoV-2 RBD accounted for 90% of serum neutralizing the activity in convalescent patient sera (40).

Grifoni and colleagues identified circulating SARS-CoV-2–specific CD8+ and CD4+ T cells in 70 and 100% of patients, respectively (41). Moreover, a robust CD4+ T-cell response to the SARS-CoV-2 spike protein was observed, which correlated with the magnitude of the anti–SARS-CoV-2 IgG and immunoglobulin A (IgA) titers (41).

## Initial or Acute-Phase Immune Response

Antibody responses are triggered by the initial interaction between the SARS-CoV-2 spike protein and the human ACE2 receptor and subsequent cellular uptake of the virus (33–35). The immune response kinetics, magnitude, and relationship to disease severity during this acute-phase response have been defined extensively. SARS-CoV-2 elicits humoral and cellular immune responses; within 7 days of infection, virus-specific memory CD4+ and CD8+ T cells emerge, peaking within 2 weeks but remaining detectable at comparatively lower levels for ≥100 days. Simultaneously, there are strong B-cell responses with immunoglobulin M (IgM) and IgA antibodies detected by days 5–7 and IgG antibodies by days 7–10 (42). The magnitude of both antibody and T-cell responses is not uniform among individuals with COVID-19 and appears to be influenced by disease severity (42–45). Antibody levels and the CD8+ T-cell response subsequently decline after the acute phase of infection (42).

Similar immune response kinetics immediately postvaccination have been observed and noted in data published from human clinical trials of late-stage COVID-19 vaccine candidates using spike protein as antigen: spike-protein–specific neutralizing antibodies were elicited by vaccination, and the titers peaked 7–14 days postcompletion of the vaccination series, and in most cases, these humoral responses were comparable with those observed in the respective panels of convalescent patient sera (46–48). Robust antigen-specific CD4+ and CD8+ T cell responses were observed with the BNT162b2 mRNA vaccine and the mRNA-1273 vaccine (47, 49, 50); notably both vaccines induce strong T-helper type 1 (Th1) CD4+ T cell responses with minimal to no T-helper type 2 (Th2) responses (49, 50). T cell-mediated responses have also been reported for 3 Ad-based vaccines: chimpanzee ChAd-Ox1 n-CoV-19, Ad5-vectored COVID-19 vaccine and recombinant (r) Ad26 and rAd5 vector-based vaccine (48, 51–53).

## Breadth of Immune Coverage of Emerging SARS-CoV-2 Variants

Like other pathogenic viruses, SARS-CoV-2 continues to accumulate mutations due to natural evolution and immune pressure as it propagates through the human population. CoV RNA replicases have been reported to be 10-fold less error-prone than other RNA viruses (54). This may explain the substantially lower sequence diversity of glycoprotein antigens like the SARS-CoV-2 spike protein compared with influenza A virus antigens hemagglutinin and neuraminidase (437-fold greater diversity), and with viruses that cause chronic infections and are influenced by immune pressure on the virus, such as HIV and hepatitis C virus (54). However, natural viral evolution during 2020 has produced novel SARS-CoV-2 variants that carry several distinct mutations in the spike protein (55), which have materially changed the COVID-19 pandemic. Key variants include the D614G spike protein mutant that has shown a modest ability for faster spread (56), and the mink variant with multiple mutations (e.g., “cluster 5”) that demonstrate spillover transmission across species and highlight the risk of incrementally evolved SARS-CoV-2 viruses with broad host range and/or greater pathogenicity (57–59). Most recently, at least 3 new variants, B.1.1.7 (first observed in the United Kingdom), B.1.351 (first observed in South Africa), and P.1 (first observed in Brazil), have arisen and are spreading across the globe (55, 60).

Questions persist regarding difference in clinical severity of COVID-19 caused by these variants and the breadth of immune coverage against these new SARS-CoV-2 variants. The D614G mutation causes an allosteric change in the spike RBD (61, 62), and exhibits efficient replication *in vitro* and transmission in animal models (63, 64); however there are no indications thus far of higher clinical severity or mortality (65). Additionally, D614G mutant pseudovirus is neutralized by both convalescent patient sera and vaccine-elicited immune sera (66, 67). Mink-associated variants are not associated with rapid spread or increased disease severity among humans, and preliminary serology assessments show that convalescent sera with varying antibody titers neutralize both wildtype and mink-variant virus suggesting that it is unlikely that these mutations will jeopardize the effects of vaccines or therapeutics (68, 69). The B.1.1.7, variant emerged quite rapidly through December 2020 in the United Kingdom (70, 71). Analysis of 228,361 secondary cases identified *via* England's National Health Service Test and Trace system between October 5 and December 6, 2020 revealed the index case was infected with B.1.1.7 in 15.1%, and other variants in 9.8% of cases (72), this 30–50% increased transmissibility is consistent with the earlier modeling data (70, 71). A matched cohort study of confirmed wildtype and B.1.1.7 cases in England (1,769 in each group) identified between September 20 and December 15, 2020 demonstrated no statistically significant difference in hospitalization, 28-day case fatality, or likelihood of reinfection (72). However, additional accumulating evidence in January and February 2021 suggests B.1.1.7 infection is associated with increased risk of hospitalization and death, though the absolute mortality risk per infection remains low (73). During this same period, a similar, but not identical, variant named 501Y.V2 (B.1.351) has become prominent in South Africa, wherein 3 of 8 defined mutations in the spike protein – N501Y, E484K and K417N – lie within the receptor-binding motif that forms the interface with the human ACE2 receptor (60, 74).

Studies have shown that certain mutations/variants (E484K mutation in particular) are able to escape neutralization by convalescent plasma and monoclonal antibodies that target single epitopes (75–77). Currently authorized vaccines and almost all late-clinical stage candidates contain the full-length SARS-CoV-2 spike protein as antigen (46–48, 51, 53), thereby targeting epitopes outside the RBD (49). The 2 mRNA vaccines have demonstrated ~95% VE against symptomatic COVID-19 and have been shown to elicit polyclonal antibodies similar to natural infection (10, 11, 78); these newly developed vaccines have demonstrated breadth of neutralizing antibody activity against assorted variants, albeit with small declines. BNT162b2 mRNA vaccine immune sera tested against isogenic wildtype and N501Y SARS-CoV-2 on a strain Wa-1 genetic background found no loss of neutralization titer against the N501Y variant strain (79). Vaccine-elicited sera were able to neutralize recombinant viruses containing key variant mutations *in vitro*: BNT162b2 induced equivalent geometric mean titers (GMTs) of neutralizing antibodies against the B.1.1.7 variant vs. the reference (Wuhan) strain (80), and showed a 0.81- to 1.46-fold reductions in GMTs against mutations in B.1.1.7 and B.1.351 variants relative to the parental virus (81); mRNA-1273 demonstrated no reduction against the B.1.1.7 variant, and a 6.4-fold reduction in neutralizing titers against the B.1.351 variant (82). Adenoviral vector vaccine ChAdOx1-nCoV-19-elicited sera showed 9-fold lower *in vitro* neutralization activity against the B.1.1.7 variant vs. a canonical non-B.1.1.7 lineage virus (83). An inactivated whole-virus vaccine, BBV152, elicited sera with comparable *in vitro* neutralization activity against a B.1.17-like virus of Indian origin vs. the homologous vaccine viral strain (84).

Finally, reduced VE against clinical disease has been observed in late-stage clinical trials in regions with high circulation of emerging variants. Interim analysis in January 2021 for an adenoviral vector vaccine (Ad26.COV2.S) (85) in a single-dose regimen reported 66% VE overall against moderate to severe COVID-19, with 72% VE in the United States and 66% VE in Latin America cohorts, but VE dropped to 57% in the South African cohort, wherein 95% of the accrued cases were caused by the B.1.351 variant (18). Similarly, phase 3 results for the adjuvanted protein subunit vaccine NVX-CoV2372 showed 89.3% VE in the UK cohort, where >50% of accrued cases were caused by the B.1.1.7 variant, while VE in the South African cohort was 49.4%, wherein 93% of the accrued cases were caused by the B.1.351 variant (23). Notably, COVID-19 cases in the placebo group accrued regardless of serologic evidence of prior SARS-CoV-2 infection – attack rate in seropositive 3.9% (26/674 cases) vs. seronegative 3.9% (58/1494 cases) (86), suggesting that B.1.351 variant could escape antibodies elicited by previous natural infection. Novel SARS-CoV-2 variants may thus warrant re-designing vaccines and/or implementing booster vaccines for the global population without immunity arising from natural infection. Regulators are collaborating with manufacturers to develop guidance for the types of data needed to support updated vaccine compositions and streamlined clinical programs that can demonstrate vaccine effectiveness expeditiously (87).

## Preexisting and Cross-Reactive Antibodies

SARS-CoV-2 is a new pathogen, and most of the global population has yet to achieve substantial immunity to it. However, this virus belongs to a known family of CoVs, and it is informative to investigate the contributions of preexisting and cross-reactive immunity, which potentially could contribute to protection against SARS-CoV-2. It is important to understand how that might affect the susceptibility to and the severity of SARS-CoV-2 infection. Although studies of convalescent patient sera showed that natural infection elicited an immune response, the fundamental evidence that natural infection with SARS-CoV-2 confers protective immunity as well as protection against reinfection was obtained from a non-human primate challenge study (88). Inoculation of 9 adult rhesus macaques with a total of 1.1 × 10^4^ to 1.1 × 10^6^ plaque-forming units (PFU, *n* = 3/group) resulted in high levels of viral RNA in both the upper and lower respiratory tracts as well as symptoms suggestive of mild clinical disease and, most importantly, induction of humoral (anti–SARS-CoV-2 spike protein) and cellular immune responses (88). On rechallenge 35 days after the initial infection, only prechallenged animals exhibited a rapid anamnestic immune response and minimal clinical disease compared with those of naive animals. Minimal to no viral RNA was detected in the respiratory tract of prechallenged animals, although viral RNA concentrations were higher in nasal swabs compared with bronchoalveolar lavage, reminiscent of disease and increased transmissibility in humans (88).

Another study profiled the immune response across multiple human CoV RBDs, respiratory viruses, and SARS-CoV-2 to determine whether heterologous immunity to other CoV-RBDs or other infections influenced the evolution of the SARS-CoV-2 humoral immune response (89). The study found little evidence of correlation between SARS-CoV-2 responses and HKU1, NL63, and respiratory infection (influenza and respiratory syncytial virus) responses, suggesting that common viral infections including common CoV immunity, targeting the RBD involved in viral infection, do not influence the rapid functional evolution of SARS-CoV-2 immunity and thus should not affect diagnostics or vaccine-induced immunity (89).

Another study characterized spike RBD antibody kinetics and isotype profile in COVID-19 cases (*n* = 343) and prepandemic controls (*n* = 1,548) evaluating antibody responses to RBDs derived from spike proteins of endemic human CoVs (HKU1, 229E, OC43, and NL63), SARS-CoV-1, and MERS (90). No cross-reactive responses to endemic human CoV RBDs or MERS-CoV RBD were seen in COVID-19 cases, but significant cross-reactivity to SARS-CoV-1 RBD was observed (90).

Individuals with high levels of T cells that are able to recognize SARS-CoV-2 could potentially mount fast and strong immune responses to reinfection, possibly limiting disease severity (91). In a study of 36 individuals recovering from mild to severe COVID-19, all had SARS-CoV-2–specific CD4+ and CD8+ T cells that recognized multiple regions of the SARS-CoV-2 nucleocapsid (N) protein (92). The same study examined T cells in 23 individuals who had recovered from SARS in 2003. Seventeen years after infection, these individuals possessed long-lasting memory T cells that were reactive to the SARS-CoV-1 N protein and also cross-reactive to the N protein of SARS-CoV-2. Similarly, Mathew and colleagues examined blood from 125 hospitalized COVID-19 patients and found that more severe COVID-19 was associated with lower frequencies of both CD4+ and CD8+ T cells (93). However, there is much evidence showing that antibodies to the spike protein rather than the N protein are responsible for neutralizing activity against SARS-CoV-2 (94).

Finally, a retrospective analysis of nearly 16,000 electronic health records from a single academic medical center of patients with and without PCR evidence of recent or ongoing infection with at least 1 of 4 seasonal endemic human CoVs demonstrated a similar propensity to acquire SARS-CoV-2 (95). These data suggested that any immunity elicited by prior CoV exposure was insufficient to prevent subsequent infection by SARS-CoV-2 but may be associated with less severe COVID-19 (95).

## Durability of Immunity

The persistence of the immune response to SARS-CoV-2 and how this might contribute to preventing reinfection is not well-understood at this stage of the pandemic. Furthermore, duration of protection is a key question for vaccine development and the need for periodic revaccination to overcome antibody waning, and has substantial implications for achieving and maintaining herd immunity.

Initial evidence for potential duration of protection from novel SARS-CoV-2 infection comes from a long-term epidemiologic study on immunity to seasonal CoVs. Edridge and colleagues followed a cohort of 10 adult men since the 1980's, evaluating immunity to seasonal CoVs—NL63, 229E, OC43, and HKU1—by measuring the frequency of reinfection, sampling every 3–6 months annually for >35 years (8). They concluded that protective immunity against seasonal CoVs is short term (i.e., lasting 6–12 months) because they observed that natural reinfections occurred for all seasonal CoVs at 6 and 9 months but most frequently within 12 months (8).

In a large US study of >30,000 individuals who tested positive for SARS-CoV-2, ~93% had moderate to high titers of antispike antibodies, with >90% of these individuals having detectable neutralizing antibody responses (96). The researchers reported that antibody titers were stable over 3 months with modest declines at 5 months; longer-term studies are needed to help establish a correlate of protection (96).

Persistent immune responses to SARS-CoV-2 have also been assessed by antibody isotype in a cohort of patients with symptomatic SARS-CoV-2 from North America (*n* = 259); IgM and IgA responses to SARS-CoV-2 RBD were transient, and most patients seroreverted within 2.5 months after symptom onset (90). However, IgG responses were sustained for ≥90 days, and seroreversion was minimal (90). Similarly, in a study of serum (*n* = 439) and saliva (*n* = 128) samples from patients with COVID-19, antibody responses to the SARS-CoV-2 spike RBD were detected in both fluids, with peak IgG levels attained by 16–30 days after symptom onset (97). IgA and IgM levels declined, while IgG antibodies remained relatively stable for ≥3 months after symptom onset. Neutralizing antibodies peaked between 31 and 45 days and then declined up to 105 days (97).

A population-based serosurveillance study (*n* = 30,576) in Iceland, where 15% of the country's citizens had been tested for COVID-19 by quantitative PCR (qPCR), showed that ≥90% (*n* = 1,107/1,215) of qPCR-positive individuals remained seropositive 120 days after diagnosis, and there were no observed declines in antibody titers (98). When these responses were broken down by isotype and antigen, IgM anti-N antibody levels were transient and no longer detectable within 2 months, whereas IgA anti-S1 antibodies declined 1 month after diagnosis but remained detectable, and IgG anti-N and anti-S1 increased within 6 weeks after diagnosis and persisted but with a slight decrease in levels thereafter (98). A longitudinal cohort study of >1,100 seropositive healthcare workers reported that previous SARS-CoV-2 infection that generated antibody responses offered protection from reinfection for most people in the 6 months after infection (99).

More recently, Dan and colleagues measured circulating SARS-CoV-2 antigen-specific antibodies, memory B cells, and CD8+ and CD4+ T cells for >6 months postinfection in a cohort of patients with COVID-19 (n = 185) across a range of disease including asymptomatic, mild (non-hospitalized), moderate (hospitalized), and severe (hospitalized) (37). Spike RBD IgG titers were almost stable from days 20 to 240 postsymptom onset (half-life [t½] = 140 days), and antigen-specific memory B cells increased over the first ~150 days and then plateaued. Half of the patients were positive for circulating SARS-CoV-2 memory CD8+ T cells at ≥6 months (t½ = 166 days), and 89% of patients were positive for circulating SARS-CoV-2 memory CD4+ T cells at ≥6 months (t½ = 150 days) (37). The authors combined 5 immune components to devise a composite measurement of SARS-CoV-2 immune memory: RBD-specific IgG, IgA, and memory B cells and SARS-CoV-2–specific CD4+ and CD8+ T cells. They found that at 1 to 2 months postinfection, 59% of individuals were positive for 5/5 components, and at ≥5 months postinfection, 40% were positive for 5/5 components; however, 96% of individuals were positive for 3/5 components (37).

For SARS-CoV-2 mRNA vaccines recently authorized for emergency use (12, 13), data on longer-term persistence of vaccine-elicited immune responses will become available as the participants from the phase 3 vaccine clinical studies are followed for 2 years postprimary vaccination (10). An interim readout of immune persistence has been reported for mRNA-1273 suggesting that antibody binding and neutralization titers were maintained for 3 months postprimary vaccination among study participants (100). An Ad vector vaccine against a related CoV, ChAdOx1 MERS, showed that vaccine-elicited neutralizing antibodies waned from peak postprimary immunization levels and remained stable above baseline for ≥1 year (101).

## Systems Serology and Systems Immunology

Systems serology is an experimental approach that aims to define comprehensively the varied humoral responses to vaccine antigen(s) by measuring both polyclonal antibody features (antigen-binding portion) and functional properties (Fc portion) and can help elucidate mechanisms of immunity, define multifactorial correlates of protection, and evaluate vaccine candidates (102). We summarize a number of promising system serology investigations that have enhanced our understanding of the distinct early immune response profiles for COVID-19, some of which could serve as biomarkers for predicting COVID-19 trajectory.

Using systems serology, Atyeo and colleagues characterized the antibody features and functions of the SARS-CoV-2–specific humoral immune response in a cohort of 22 SARS-CoV-2–positive patients (recovered, *n* = 12; deceased, *n* = 10) (103). They identified distinct humoral profiles comprising 5 antibody features that could differentiate between patients who recovered or eventually died of SARS-CoV-2 disease and could potentially be developed into a biomarker that predicts COVID-19 severity in humans (103). Specifically, convalescent and deceased patients presented distinct humoral profiles: more spike-focused response in convalescent patients and stronger N-specific responses in deceased patients. Convalescent patients showed enriched spike-specific phagocytic and complement activity, whereas those who died of disease had poorly coordinated RBD-specific antibody-dependent complement deposition and natural killer (NK) cell functions as well as a greater N-specific response (103). This early divergence (spike:N ratio) in their humoral immune response was confirmed in a larger validation cohort of acutely infected individuals (recovered, *n* = 20; died, *n* = 20) (103). Equivalent titers and functional profiles were observed across both groups, indicating that the magnitude of the responses was comparable and did not contribute to the observed differences in either population (103).

Potent neutralizing antibodies against SARS-CoV-2 appear to increase survival and may protect against reinfection with variants of SARS-CoV-2. In a cohort of 113 patients infected with SARS-CoV-2 stratified by disease severity and outcomes (i.e., non-hospitalized, hospitalized, intubated, immunosuppressed, and deceased), anti-RBD antibody levels, neutralization titers, and neutralization potency indices predicted disease severity and survival (66). Comprehensive immunophenotyping of peripheral blood in 42 individuals with divergent clinical trajectories of SARS-CoV-2 infection and COVID-19 (moderate *n* = 7, severe *n* = 28, recovered *n* = 7) showed perturbations in multiple leukocyte populations, which distinguished cases of severe COVID-19 from healthy donors, cases of moderate COVID-19, and from patients who have recovered from COVID-19 (44). This distinct immune phenotype included activation of CD4+ and CD8+ T-cell memory subsets, substantial oligoclonal expansion of B cells, and dysregulation of innate immune subsets (44). Moreover, these analyses further confirmed the prognostic value of the neutrophil to lymphocyte ratio as a biomarker of disease severity and organ failure. Similarly, longitudinal immune profiling of peripheral blood mononuclear cells and plasma samples from 113 patients with moderate (*n* = 80) or severe (*n* = 33) COVID-19 identified 4 distinctive immune response profiles that predicted divergent disease courses within 10 days of infection (104). Patients with severe COVID-19 and poor clinical outcome showed an aberrant immune response that included an early elevation in proinflammatory cytokines. In all patients, an overall increase in innate cell lineages such as monocytes, low-density neutrophils, and eosinophils was seen, with a parallel decrease in CD4+ and CD8+ T-cell levels (104). A study by Mathew and colleagues found 3 distinct immunotypes in hospitalized COVID-19 patients: (1) robust activation of CD4+ T cells and highly activated or exhausted CD8+ T cells, (2) less robust CD4+ T cells and highly functional effector-like CD8+ T cell responses, and (3) a lack of lymphocyte response (93). These major patterns may represent different suboptimal responses to infection (93).

In a study from Spain, investigators observed that plasma SARS-CoV-2 RNAemia and viral RNA load were significantly correlated with severe illness in COVID-19 (105). Among patients with COVID-19, 78% of critically ill patients had a significantly higher frequency of plasma SARS-CoV-2 RNAemia compared with 27% of ward patients and 2% of outpatients (*P* < 0.001) (105). Viral RNA load in plasma was also associated with a maladaptive immune response profile that included higher levels of chemokines, NK cell activation as well as evidence of a systemic inflammatory response (e.g., IL-6), endothelial dysfunction, coagulation activation, and tissue damage (105). Another study also reported the presence of activated NK cells with upregulated levels of chemokines and effector molecules, nutrient receptors, and activating receptors in persons with moderate to severe COVID-19 (106).

Time to seroconversion may also be predictive of more severe disease. In 159 symptomatic patients with COVID-19 from North America, the median time to seroconversion to SARS-CoV-2 RBD was, on average, 4 days earlier for hospitalized patients compared with non-hospitalized patients over all isotypes (IgG, IgM, and IgA), suggesting an association between antibody kinetics and disease severity (90).

Collectively, the evidence from these studies suggests that uncontrolled viral replication coupled with a reduced innate antiviral defense and a maladaptive proinflammatory response are intrinsic elements to the pathogenesis of COVID-19 (105, 107). Further research is needed to identify which biomarkers may predict those at risk of severe disease ideally to determine a limited number of readily measured markers that are useful in predicting clinical outcomes (45).

## Immunity in Special Populations

At this stage in the pandemic, there is limited evidence regarding the immune response to COVID-19 during pregnancy, postpartum, breastfeeding, and in neonates. None of the completed or ongoing phase 3 clinical studies of vaccines include pregnant women (108), and further research is needed to understand the immune response in these special populations.

A study of immune responses in pregnant and non-pregnant women who either tested positive (*n* = 22) or negative (*n* = 11) for SARS-CoV-2 before delivery, found that pregnant and non-pregnant women showed similar IgG responses recognizing the full-length SARS-CoV-2 spike protein, whereas pregnant women had significantly lower anti–spike-RBD IgG titers than did non-pregnant women (108). Pregnant women were also less likely to have detectable neutralizing antibody titers (108). Studies of maternal and cord blood serum found that maternal transfer of neutralizing antibodies may be reduced, but SARS-CoV-2 infection did not generally affect maternal transfer of humoral immunity, as measured by placental Fc receptor and maternal and cord blood serum anti-tetanus IgG titers (108).

A study in the United States found no SARS-CoV-2 RNA detected in breast milk collected from 18 women with mild to moderate COVID-19 (109), however, breast milk did contain anti–SARS-CoV-2 antibodies (IgA and IgG), and concentrations correlated with virus neutralization titers (109). These results are supported by another study in China in 14 women in whom no SARS-CoV-2 RNA was found in breast milk, but in this study, SARS-CoV-2 antibodies (IgM or IgG) were found in only 3 samples (110). Both studies recommended that breastfeeding should continue with care in women infected with SARS-CoV-2 (109, 110). Another study in China investigated the presence of SARS-CoV-2 antibodies in 64 infants, of whom 24 were born to mothers with confirmed COVID-19. Among these 24 infants, 15 had detectable IgG antibodies and 6 had IgM antibodies; none had positive nucleic acid test results (111). IgM and IgG titers gradually declined in all infants; however, IgG titers had a slower decline in infants with positive IgM results (111). Further research is needed to determine a correlation of immunity in infants with antibodies to SARS-CoV-2 (111).

In a study conducted in the United Kingdom, the kinetics of SARS-CoV-2 antibody responses was investigated by measuring seroprevalence in 849 children aged 2–15 years (112). Sixty-five children (7.7%) were positive when first tested, and 45 of those had detectable antibody titers when tested ~2 months later. A systematic review of SARS-CoV-2 in pediatric populations reports that while children have lower case rates compared with adults, they may be just as susceptible to infection but have fewer symptomatic infections (113). While it is not fully understood why children appear to have milder illness compared to adults, it has been suggested that this may be partly due to trained immunity due to routine live childhood vaccinations and frequent viral infections (114). However, additional rigorous studies are essential to better understand the clinical course, transmission, and immune response to COVID-19 in children (113, 114).

Among the 2 mRNA vaccines recently authorized for emergency use (12, 13), BNT162b2 mRNA vaccine is authorized for individuals 16 years and older and is being evaluated in children 12–15 years of age (115). In addition, the mRNA-1273 vaccine is authorized for individuals 18 years and older (13), and is being evaluated among children 12–17 years of age (116).

## Discussion

**Figure 1:**
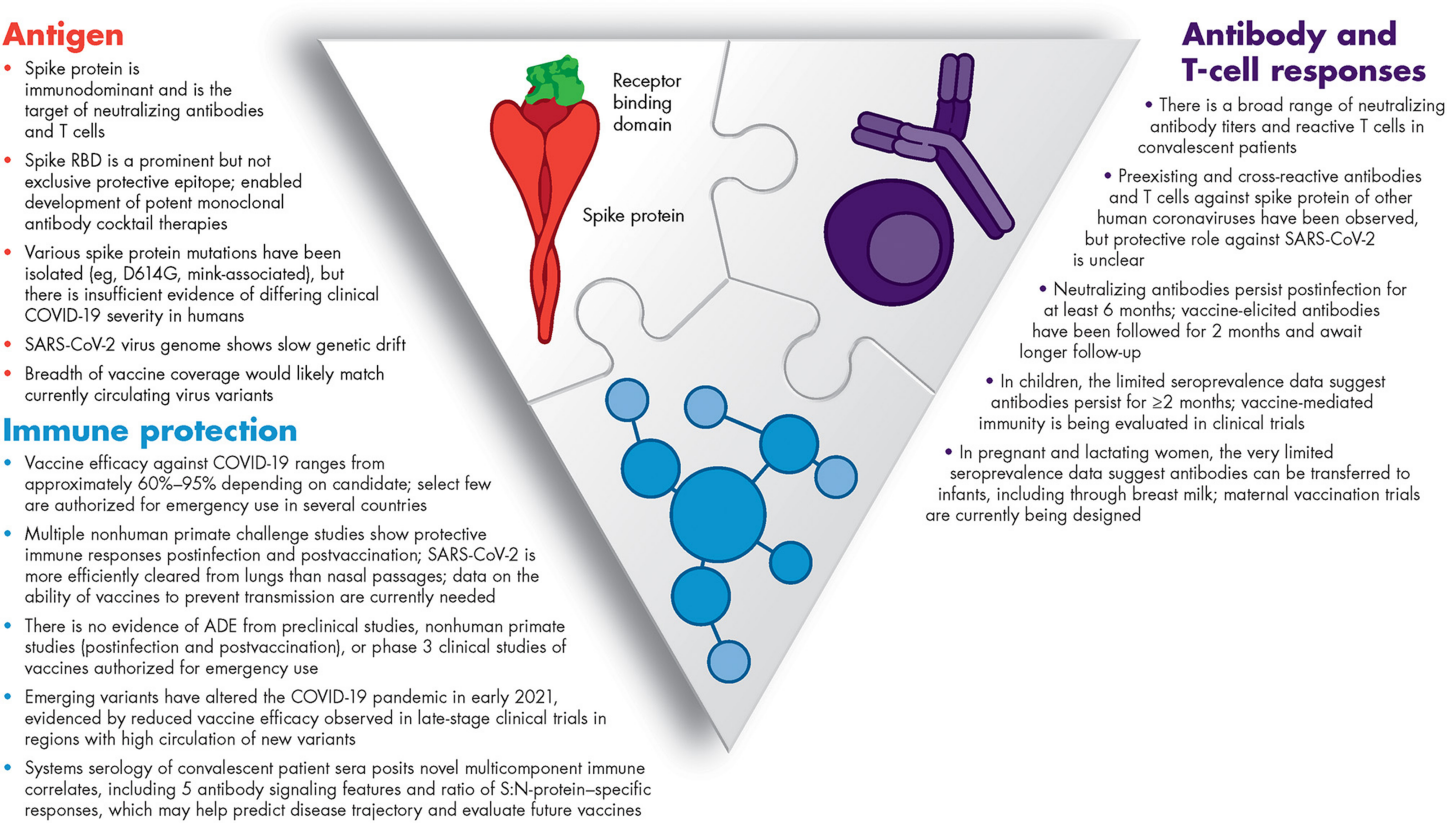
Summary of our current knowledge of immunity to SARS-CoV-2. ADE, antibody-dependent enhancement; COVID-19, coronavirus disease 2019; RBD, receptor-binding domain; SARS-CoV-2, severe acute respiratory syndrome coronavirus 2.

Unprecedented collaborative research among the global scientific and medical community continues to rapidly advance our understanding of COVID-19 and the associated immune response. This emerging knowledge enabled the development of immunotherapeutics and vaccines. Figure 1 summarizes what we have learned in <1 year about the SARS-CoV-2 antigens and humoral and cellular immunity, and what we have learned from both in terms of predicting COVID-19 trajectory (biomarkers) and vaccine efficacy (immune correlates). Yet, there is still much to understand about this novel virus – SARS-CoV-2.

An effective vaccine against SARS-CoV-2 is thought likely to be the best strategy to control the current COVID-19 pandemic (117), and as of writing, 2 mRNA vaccines have been granted EUA based on ~95% vaccine efficacy against COVID-19 (10, 11) and prioritized individuals are being vaccinated in stages in several countries across the globe, marking a key turning point in this pandemic. Several other candidates—notably viral vector and inactivated virus vaccines—are in late stage clinical trials and are anticipated to become available in the coming months and improve the global vaccine supply (118). In addition to vaccines, neutralizing mAbs to SARS-CoV-2 isolated from convalescent patient sera have been developed, and 2 mAb therapies have been granted EUA (28, 29, 119). These mAbs can be deployed in acute situations to block disease progression and protect those exposed or unvaccinated in high risk settings, or individuals who may be allergic or unlikely to respond to a vaccine (119). In these settings, early and rapid testing of individuals at high risk of infection have been suggested (120). Phase 3 study results report that REGN-COV2, a mAb cocktail, reduces viral load with the most benefit observed in patients with high viral loads at baseline and in those who had not yet initiated an immune response (i.e., those positive for SARS-CoV-2 but serum antibody-negative) (121). In contrast, a study of mAb LY-CoC555 did not show any benefit in patients hospitalized with COVID-19 (122). More research is required to fully realize the potential of mAb therapy in COVID-19.

A major role of neutralizing antibodies is binding of antigens as well as interaction with cells with Fc receptors to induce Fc effector functions (4, 123). There is a potential risk, however, of antibody-dependent enhancement (ADE) of disease mediated by these Fc functional antibodies (4, 123). This phenomenon has been observed with SARS-CoV-1 (123). It is important to understand the balance between Fc signaling that promotes protective immunity and that which promotes inflammatory pathology, and whether systems serology can identify those at risk (124). To date, there has been no evidence of ADE due to SARS-CoV-2 vaccines from preclinical studies or non-human primate studies (125–128). Similarly, no evidence of ADE emerged in a phase 3 clinical study of an mRNA vaccine against SARS-CoV-2 after a median 2 months of follow-up (10). While rare anaphylactic reactions to both mRNA vaccines have been reported (between 2.5–4.7 cases per million doses of vaccine administered) (129), vaccination, along with specified postvaccination observation periods, continues to be recommended except in individuals with history of severe/immediate allergic reaction to previous mRNA vaccines or immediate allergic reaction to any of its components (130).

For many different viral infections, such as influenza, measles, and hepatitis A and B viruses, correlates of protection have been established; these are usually based on the level of antibody that is acquired from vaccination or natural infection that is able to significantly reduce the risk of infection or reinfection (96). However, correlates of protection have yet to be defined for COVID-19 (4, 42, 90). Immunogenicity data from longer term follow-up of vaccine phase 3 trial participants should be helpful to define immune correlates, especially in the case of the vaccines that have already achieved emergency authorization for pandemic use (12, 13). In addition, systems serology studies might yield novel immune correlates based on composite antibody and cellular immune measurements. Regardless, immune correlates will catalyze rapid vaccine development for special populations such as pediatric populations, pregnant and lactating women, and those with immunosuppressive conditions, where traditional field efficacy trials would be unfeasible, and potentially for vaccines with updated compositions to address novel SARS-CoV-2 variants.

## Author Contributions

All authors contributed to the conception, design of the article, interpreting the relevant literature, drafting of the manuscript, and/or critically revising it for intellectual content.

## Conflict of Interest

The authors declare that this study received funding from Pfizer Inc. The funder had the following involvement in the study: Editorial/medical writing support. JF is a speaker for Pfizer and a consultant/advisory board member for Pfizer and GlaxoSmithKline. AS is a Pfizer employee and may hold stock or stock options.
